# Supplementary material for: Disalicylic Acid Provides Effective Control of Pectobacterium brasiliense
Source: Microorganisms. 2022 Dec 19;10(12):2516. doi: 10.3390/microorganisms10122516 (PMC9784377; doi:10.3390/microorganisms10122516)
Supplement: Supplementary file 1 [file microorganisms-10-02516-s001.zip › microorganisms-2083216-supplementary.pdf]

Supplementary Figure

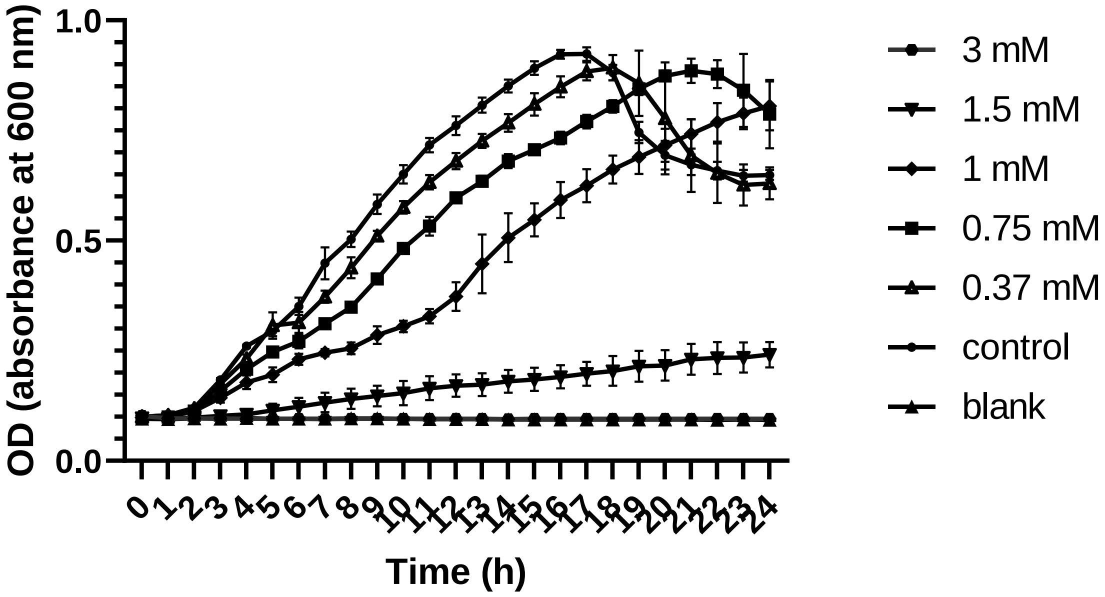

Figure S1. Growth of Pb1692 in the presence of different concentration of DSA. Absorbance at 600 nm was measured once every hour for 24 h. DSA was dissolved in LB. Bacteria were grown at 28°C for 24 h (bar = SE; *n* = 4).
